# Octopamine Neuromodulatory Effects on a Social Behavior Decision-Making Network in *Drosophila* Males

**DOI:** 10.1371/journal.pone.0013248

**Published:** 2010-10-12

**Authors:** Sarah J. Certel, Adelaine Leung, Chih-Yung Lin, Philip Perez, Ann-Shyn Chiang, Edward A. Kravitz

**Affiliations:** 1 Department of Neurobiology, Harvard Medical School, Boston, Massachusetts, United States of America; 2 Brain Research Center, National Tsing Hua University, Hsinchu, Taiwan; 3 Division of Biological Sciences, University of Montana, Missoula, Montana, United States of America; 4 Institute of Biotechnology, National Tsing Hua University, Hsinchu, Taiwan; University of California Davis, United States of America

## Abstract

Situations requiring rapid decision-making in response to dynamic environmental demands occur repeatedly in natural environments. Neuromodulation can offer important flexibility to the output of neural networks in coping with changing conditions, but the contribution of individual neuromodulatory neurons in social behavior networks remains relatively unknown. Here we manipulate the *Drosophila* octopaminergic system and assay changes in adult male decision-making in courtship and aggression paradigms. When the functional state of OA neural circuits is enhanced, males exhibit elevated courtship behavior towards other males in both behavioral contexts. Eliminating the expression of the male form of the neural sex determination factor, Fruitless (Fru^M^), in three OA suboesophageal ganglia (SOG) neurons also leads to increased male-male courtship behavior in these same contexts. We analyzed the fine anatomical structure through confocal examination of labeled single neurons to determine the arborization patterns of each of the three Fru^M^-positive OA SOG neurons. These neurons send processes that display mirror symmetric, widely distributed arbors of endings within brain regions including the ventrolateral protocerebra, the SOG and the peri-esophageal complex. The results suggest that a small subset of OA neurons have the potential to provide male selective modulation of behavior at a single neuron level.

## Introduction

The processes of encoding, decoding, rapidly interpreting, and appropriately responding to information derived from complex surroundings are essential for the reproductive success and survival of organisms. How sensory input, internal physiological state, and individual experience interact in the gathering and utilization of information in deciding on behavioral responses are key issues in understanding nervous system function. Studies of simple neural networks such as the lobster stomatogastric nervous system, the *Aplysia* gill withdrawal reflex, and the pond snail feeding system provide fundamental insight into how circuits function to elicit rhythmic or reflexive behaviors (reviewed in [1], [2]). Yet in terms of whole organism behavior, far less is known. Detailed studies of olfactory-mediated behavior in *Drosophila* are beginning to unravel receptor-sensory neuron-interneuron connections in fly brains. Even here though, the circuitry remains largely undefined beyond the second-order projection neurons [3], [4], [5], [6]. Moreover, complicating straightforward linear connectivity analyses of the latter types is the knowledge that the output of behaviorally relevant circuitry can be changed by modulatory neurons [7], [8]. Neuromodulators released by amine- and peptide-containing neurons are evolutionarily conserved and broadly utilized within the animal kingdom. Prominent among these substances in vertebrate systems are the amines norepinephrine, serotonin and dopamine.

At a cellular level, neuromodulators can regulate excitatory and inhibitory synaptic transmission and neuronal firing levels with modulatory neuron firing often maintained through feedback mechanisms mediated by autoreceptors [9], [10], [11], [12]. The temporally slow, spatially diffuse, and long lasting effects of neuromodulators complicate understanding their influence on network dynamics. Recent computational models suggest that norepinephrine and acetylcholine function to generate behavioral confidence about sensory cues guiding action selection [13]. Norepinephrine also has been suggested to optimize decision-making in multilayered networks [14], [15]. In this article, we describe two ethologically relevant experimental situations that require rapid decision-making by *Drosophila* males in the choice between courtship and aggression. We examine the patterns seen during the display of sexually dimorphic behaviors to ask how circuit modulation by the amine octopamine (OA, the invertebrate structural analogue of norepinephrine) might alter action selection by male flies responding to varying contextual stimuli.

Recent studies have shown that single or small groups of neuromodulatory neurons can exert defined, specialized, and often subtle effects on nervous system function [16], [17], [18], [19], [20]. For the approximately 100 OA-containing neurons found in the *Drosophila* nervous system, wide ranging effects on behavior have been reported, including actions on ovulation, aggression, sleep, and learning and memory [20], [21], [22], [23], [24], [25], [26]. In previous studies we reported that by reducing or eliminating OA in fly brains or by feminizing OA/Tyramine (TA) neurons through expression of a sex determination pathway gene, *transformer*, we altered decision making at a choice point between aggression and courtship in chambers designed for aggression studies, and we increased male:male courtship behavior in chambers designed for courtship competition assays [27]. We also reported that a limited number of subesophageal ganglion (SOG) neurons co-express OA and the male form of Fruitless (Fru^M^), a key regulator of male sexual behavior, and described the morphological features of one of these neurons.

Here we extend our previous behavioral and anatomical findings by utilizing several different experimental approaches to: (i) selectively enhance, rather than reduce OA neuronal function; and (ii) precisely alter the sex of a subpopulation of these neurons; (iii) describe the morphological profiles of the three Fru^M^/OA neurons in 3-dimensions. To increase rather than decrease the physiological activity of OA neurons, we expressed the thermosensitive dTrpA1 ion channel [28] selectively in OA/TA neurons. Next, we eliminate Fru^M^ expression specifically in Fru^M^/OA neurons using an RNAi approach [29]. In our earlier studies, production was blocked indirectly through *transformer* expression, an approach that potentially changes the expression of two transcription factors, Fruitless and Doublesex, and that also potentially feminizes the total population of OA/TA neurons [27]. By using an RNA-mediated interference transgene, we examine the behavioral consequences of removing Fru^M^ only in the three Fru^M^/OA neurons identified previously. Finally, to begin to ask how individual neuromodulatory neurons might function within social behavior networks, we utilized a high-resolution 3D mapping approach and examined the arborization patterns of each of the three Fru^M^/OA neurons projected onto a ‘standard’ adult male brain. Identification of potential targets of these neurons within distinct neuropil regions should provide valuable clues towards understanding how Fru^M^/OA neurons function within neuronal frameworks to mediate the choice between courtship and aggression in adult male flies.

## Results

### Activation of OA neurons increases uncertainty in behavioral object choices

To ask whether activation of OA neurons changes the choice between aggression and courtship, we directed expression of the *Drosophila* heat-activated dTrpA1 channel [28] in the OA/TA neuronal population using the *dTdc2(tyrosine decarboxylase2)-Gal4* line [30]. This thermosensitive ion channel is activated by moderate warming to 26.5°C, minimizing non-specific high temperature effects, while at the same time allowing us to enhance the activity of OA neurons with temporal specificity in adult flies. Control and OA-neuron-activated males were tested in a “competitive courtship choice” assay in which two males of the same genotype are placed together with one Canton S virgin female. As in the previous study we used intact animals, rather than headless targets, to provide the sensory and feedback cues likely to be important in decision-making [31], [32] and separate studies from our laboratory demonstrate that males do not direct aggression against headless control males (Fernandez and Kravitz, unpublished). These experiments asked whether control males (*UAS-dTrpA1/+*) or males with activated OA neurons (*dTdc2-Gal4/UAS-dTrpA1*) exhibited greater courtship preferences for males or females. We measured courtship preference by scoring the time a male spends performing wing extensions/singing towards the female or the other male during 10 min of interaction as this motor pattern is unambiguously distinguishable as courtship behavior (see Materials and Methods; [27]). In addition, to eliminate possible non-specific Gal4 or UAS P-element insertional effects, two independent *UAS-dTrpA1* and *dTdc2-Gal4* lines were used (see Materials and Methods, [27], [28]. The data gathered from different chromosomal combinations were indistinguishable from each other and therefore the results were pooled.

**Figure 1:**
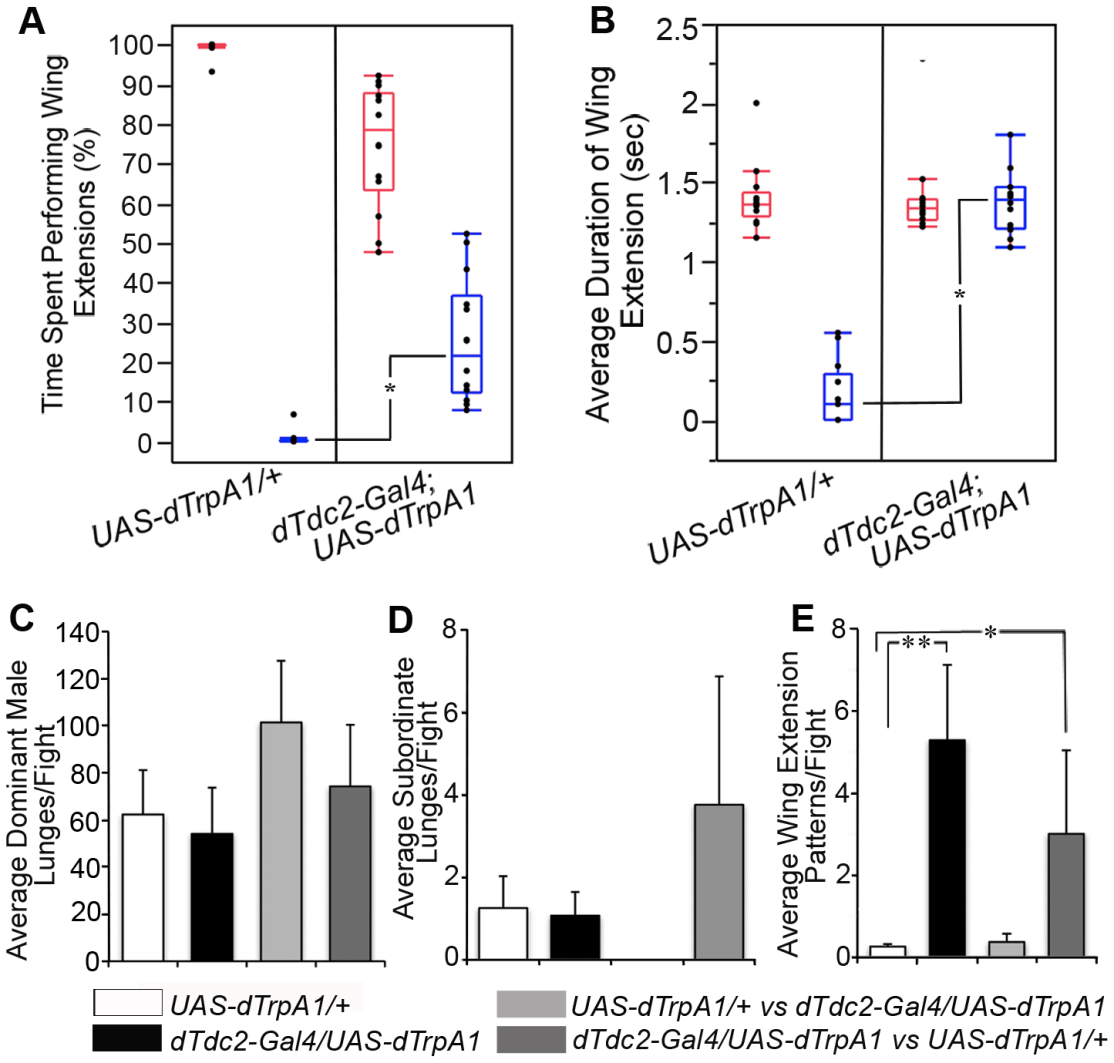
OA-neuron activated males display significant male-male courtship in competitive courtship and aggression assays. (**A**) We quantified the total time a male performed wing extensions to a female or second male during a 10 min interval in the competitive courtship assay. The box plot depicts the percentage of total time spent performing wing extensions to females (red) and males (blue). The upper and lower edges of each box correspond to the 25% and 75% quantiles. The median (50% quantile) is shown as a horizontal line in each box and the lines depict the 5% and 95% quantiles. Asterisks show medians that are statistically different according to Wilcoxon rank sum for nonparametric data: *UAS-dTrpA1/+− dTdc2-Gal4/UAS-dTrpA1 (P<.0001)*. Seven assays were performed with the *Tdc2-Gal4/UAS-dTrpA1*(III) and seven assays with the *dTdc2-Gal4#1*(new insertion)/*UAS-dTrpA1*(II) males (n = 14). Results were not statistically different and the data were pooled. (**B**) Box plot of the wing extension duration (in seconds) displayed by one male to a second fly (females indicated in red, males indicated in blue). The data were calculated by dividing the total wing extension time by the number of bouts for each male. The duration of the male-male wing extension by *UAS-dTrpA1/+*control males (n = 15) is significantly shorter than male-female wing extensions performed by the same males (Student's t test, *P<.0001*) and male-male wing extensions performed by *dTdc2-Gal4/UAS-dTrpA1* males (asterisks: Student's *t* test, *P<.0001*) (n = 14). The wing extension duration data were collected from the competitive courtship assays described above. (**C**) The graph depicts the average number of lunges the dominant male in a pairing performed per fight. There are no significant differences between controls or OA-neuron activated males in the various pairings tested. (ANOVA for independent groups, *P<0.4*). Data from the individual columns represents distinct pairings; the white column  =  two *UAS-dTrpA1/+* control males (n = 16), the black column  =  two *dTdc2-Gal4/UAS-dTrpA1* males (n = 17), the light gray column  =  one *UAS-dTrpA1/+* male and one *dTdc2-Gal4/UAS-dTrpA1* male with the *UAS-dTrpA1/+* fly emerging as the dominant male (n = 11/15 pairings), and the dark gray column  =  one *UAS-dTrpA1/+* male and one *dTdc2-Gal4/UAS-dTrpA1* male with the *dTdc2-Gal4/UAS-dTrpA1* fly establishing dominancy (n = 4/15 pairings). (**D**) The average number of lunges the ultimate subordinate male performs per fight is depicted in this graph. There are no significant differences between control males or OA-neuron activated males in the tested pairings (ANOVA for independent groups, P<0.16). (**E**) OA-neuron activated males display courtship patterns toward a second male in aggression contexts. The average number of wing extensions per fight is significantly higher in *dTdc2-Gal4/UAS-dTrpA1* male pairings than controls. (A negative binomial model (with log link function) was utilized. A likelihood ratio test of the equality of the group means yielded a highly statistically significant result (P<0.00001). Comparisons of groups yielded statistically significant differences between *dTdc2-Gal4/UAS-dTrpA1* male pairings and control *UAS-dTrpA1/+* males (Bonferroni-adjusted P = 0.00014) and between the dark gray column (one *UAS-dTrpA1/+* male and one *dTdc2-Gal4/UAS-dTrpA1* male with the *dTdc2-Gal4/UAS-dTrpA1* fly establishing dominancy) and control *UAS-dTrpA1/+* males (white column) (P = 0.0054)).

Examining multiple parameters of male courtship behavior indicated that total courtship activity was not significantly altered in OA-neuron-activated flies. The percentage of the 10 min assay time spent performing wing extensions was unchanged between control males and experimental males [*UAS-dTrpA1/+*, 16.36%±1.9 (±SEM) (n = 15) versus *dTdc2-Gal4/UAS-dTrpA1*, 16.38%±1.2 (±SEM) (n = 14); *P*>0.99, *t* test for independent samples). Although courtship initiation by experimental males (*Tdc2-Gal4/UAS-dTrpA1*) towards females was more rapid than controls, successful copulation rates were not significantly different (Table S1). Like control males, OA-neuron-activated males also spent more time singing to females than to males (Fig. 1A). However, highly significant differences were seen in the percentage of time in which *Tdc2-Gal4/UAS-dTrpA1* experimental males courted other males rather than females (Fig. 1A, Movies S1, S2, S3). Control males directed wing extensions to another male 0.7% (±0.5) of the total courtship time, whereas males with activated OA neurons courted the second male 25% (±4) of the total courtship time (Fig. 1A).

A second feature that distinguishes the courtship behavior of male *dTdc2-Gal4/UAS-dTrpA1* flies from controls is the average duration of individual bouts of male-female (M/F) and male-male (M/M) singing. When courted by other males, control males show rejection behavior, including vigorous wing flicking directed at the courter [32], [33]. This and other sensory cues usually lead to a rapid termination of all aspects of M/M courtship behavior in controls, including singing (average duration of M/M singing bouts  = 0.14 s±0.05; average M/F singing bouts  = 1.4 s±0.06, Fig. 1B). In experimental flies with activated OA neurons, however, the average duration of wing extension in M/M and M/F singing bouts were essentially identical and at levels normally seen during M/F courtship behavior (*dTdc2-Gal4/UAS-dTrpA1*, 1.37 s±0.05 vs 1.40 s±0.07, Fig. 1B). Thus both M/M courtship behavior and individual bouts of singing are extended when OA neurons are activated in this dynamic situation involving a behavioral choice between live male and live female mating partners.

### Courtship patterns interrupt aggression displays in males with activated OA neurons

In our standard aggression assay chamber, a food-filled vial cap is provided as a territory to compete over and either a headless mated female or fresh yeast paste are added as potential desirable resources within that territory [34]. In these experiments, we utilize the yeast paste resource, which should produce only aggressive responses when two socially naïve male flies are paired. In a previous report, increasing the activity of octopaminergic neurons via expression of the NaChBac sodium channel enhanced aggression above a low level seen after 5 days of group-rearing of the flies [20]. Since group reared flies are a heterogeneous population of winners and losers of multiple contests, and since fighting and decision making alters behavioral strategies [35], we used socially naïve flies as a starting population for studies in both the aggression (larger chamber with food cup and resources) and the courtship choice (smaller chamber, no food cup) experimental contexts. For studies of OA neuron activated males in an aggression setting, we paired *dTdc2-Gal4/UAS-dTrpA1* males with controls (*UAS-dTrpA1/+* parent lines) or with second OA neuron-activated males. The results show that *dTdc2-Gal4/UAS-dTrpA1* males engage in similar numbers of encounters (meetings between the flies), establish hierarchical relationships and on average, perform similar numbers of specific aggressive patterns (data not shown). We also separately examined lunges by winners and losers of fights as these dramatically differ in number from each other (winners, ca. 60/fight; losers, ca. 2–4/fight, Fig. 1C,D) and once again saw no significant effect of enhancing activity in the OA-neuron population. A small effect on the outcome of fights was seen when OA-neuron activated males were paired with control males (experimental males lost 11/15 fights). As OA-neuron activated males exhibit similar levels of aggression when paired with each other, further experiments need to be performed to determine if experimental males lose more often to control males.

We reported previously that in male flies with reduced levels of OA the likelihood of transitioning to attempted copulation after a vibrating wing extension was significantly increased [27]. Several distinct behavioral patterns involving wing usage are seen during fights between wild-type male flies including: (a) short rapid *wing flicks* utilizing one or both wings; and (b) an extended *wing threat* behavior directed at one male by the other in which “one fly quickly raises both wings to a 45° angle towards an opponent” [34], [36], [37], [38]. The behavioral pattern described previously [27], is again defined here as a 90° vibrating unilateral wing extension followed by abdominal bending and attempted copulation, instead of by a lunge. It is clearly distinguishable from *wing flicks* and *wing threats*, which usually involve both wings. In these experiments, as in our earlier studies, we observe the transition to courtship behavior mainly in males with altered OA neurons (in this case, by activation of OA neurons). We score this behavioral pattern only when it occurs on the food cup in order to clearly observe the abdominal bending following the wing extension (Fig. 1E and Movies S4, S6). This may underestimate the frequency of occurrence of the pattern during M/M fights as interactions also take place off of the food cup.

Males with activated OA neurons perform the wing extension – attempted copulation behavioral pattern on average 5.3 times per 30-minute fight in pairings of males of the same genotype, and the pattern is displayed throughout the fight by both the ultimate winner and loser males. *dTdc2-Gal4/UAS-dTrpA1* males also display the wing extension-attempted copulation pattern towards control males but with slightly reduced frequency (mean of 3 per fight) most likely due to the retreat behavior exhibited by the OA-neuron-activated males. These results, in conjunction with those of our earlier studies, suggest that without proper modulation by OA neurons, aspects of courtship circuitry can be activated within the dynamics of aggressive interactions.

### Eliminating Fru^M^ function in a small subset of OA neurons alters male action selection

**Figure 2:**
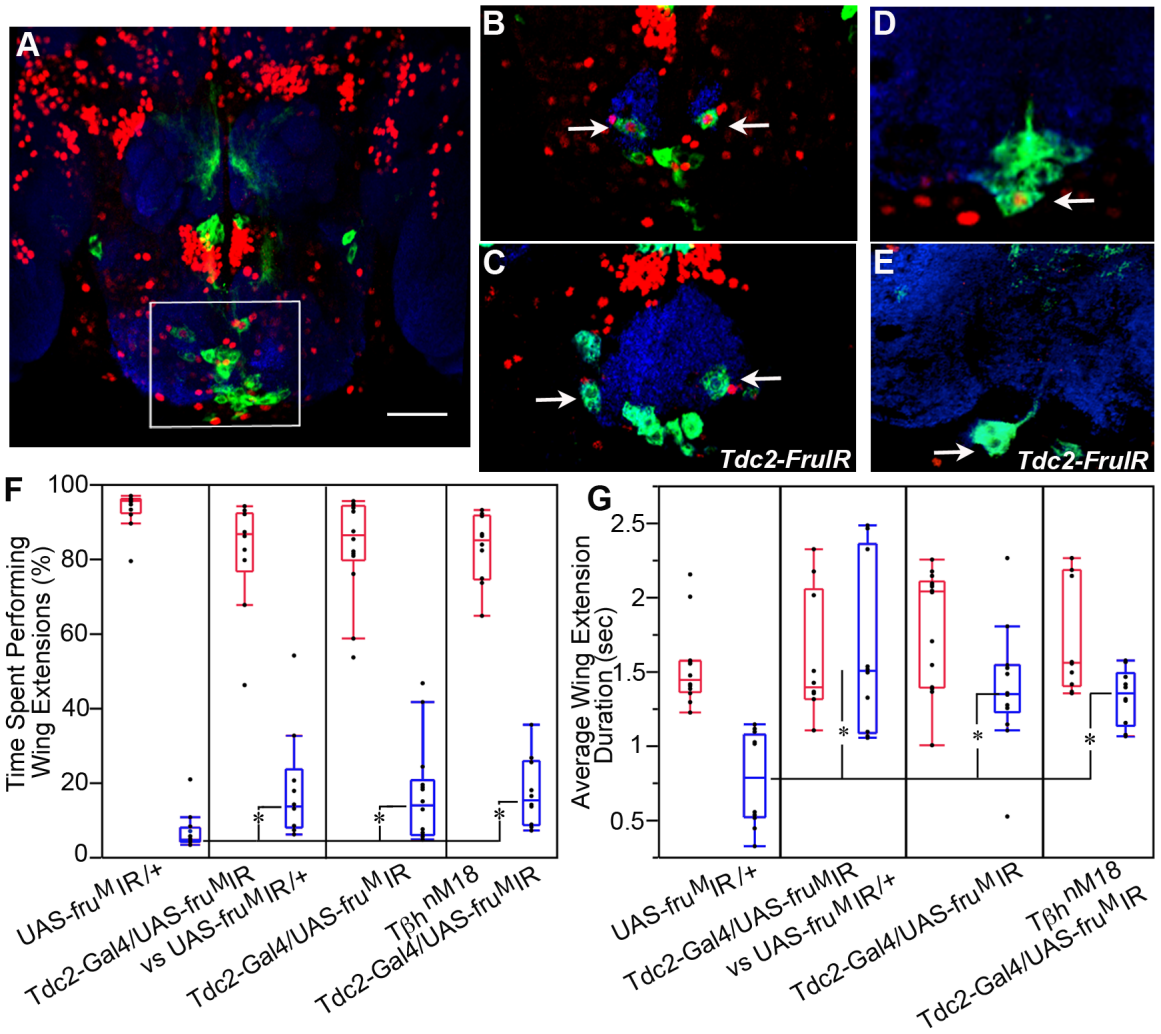
Elimination of Fru^M^ in three OA SOG neurons increases male-male courtship. Confocal sections of a transgenic *dTdc2-Gal4/UAS-mCD8:GFP* adult male brain labeled with anti-GFP (green), mAb nc82 (labels neuropil regions, blue), and anti-Fru^M^ antiserum (red), (n = 22). The SOG region contains neurons that co-express OA and Fru^M^ as highlighted by the white box. This region is the focus of the images found in B–E. scale bar  = 0.3 cm (**B**) Two ventral paired median neurons (OA-VPM, arrows) express Fru^M^ in this male *dTdc2-Gal4/UAS-mCD8:GFP* adult brain. (**C**) Fru^M^ expression is eliminated in the VPM neurons (arrows) in *dTdc2-Gal4/UAS-fru^M^ IR/UAS-mCD8:GFP* males. (**D**) A single OA-VUM (OA-ventral unpaired median neuron) neuron also expresses Fru^M^ (arrow). (**E**) Elimination of Fru^M^ OA-VUM expression (arrow) in a male *dTdc2-Gal4/UAS-fru^M^ IR/UAS-mCD8:GFP* adult brain. (n = 10 per genotype) (**F**) The box plot depicts the percentage of total time spent performing wing extensions to females (red) and males (blue). The first section is data from two control *UAS- fru^M^ IR/+* males. The second part corresponds to the courtship by a *dTdc2-Gal4/UAS- fru^M^ IR* male paired with a control *UAS- fru^M^ IR/+*male. The third plot represents data from pairings with two *dTdc2-Gal4/UAS- fru^M^ IR* males. The fourth group is data from two *Tβh^nM18^ dTdc2-Gal4/UAS- fru^M^ IR* males. The asterisks represent medians that are statistically different according to Wilcoxon rank sum for nonparametric data: *UAS- fru^M^ IR/+*− *dTdc2-Gal4/UAS- fru^M^ IR* (second section, *P<.004*), *UAS- fru^M^ IR/+*− *dTdc2-Gal4/UAS- fru^M^ IR* (third section, *P<.009*), *UAS- fru^M^ IR/+*− *Tβh^nM18^ dTdc2-Gal4/UAS- fru^M^ IR* (*P<.0015*) (n =  at least 10 for each genotype) (**G**) Box plot of the wing extension duration (in seconds) displayed by one male to a second fly (females indicated in red, males indicated in blue). The duration of the male-male wing extension by *UAS- fru^M^ IR/+*control males is significantly shorter than male-female wing extensions performed by the same males (Student's *t* test, *P<.0001*) and male-male wing extensions performed by *dTdc2-Gal4/UAS- fru^M^ IR* males paired with controls or two *dTdc2-Gal4/UAS- fru^M^ IR* males paired or *Tβh^nM18^ dTdc2-Gal4/UAS- fru^M^ IR* males (asterisks: ANOVA for independent groups, *P<.0002*). The wing extension duration data were collected from the competitive courtship assays described in (F).

Of the approximately 100 OA neurons found in the brains of male *Drosophila* [30], [39], [40], [41] we previously reported that three co-express the male form of Fruitless (Fru^M^) ([27] and Fig. 2A,B,D). Feminizing OA/TA neurons by expressing *transformer (tra)* in the *Tdc2-Gal4* driven population of neurons, yielded a phenotype in which male flies displayed courtship behavior patterns toward a second male under both aggression and courtship choice assay conditions. Since Tra is involved in the splicing of two sex determination factors, *fru* and *doublesex (dsx)* to male and female forms (reviewed in [42], [43], we asked whether Fru^M^ function is specifically required in this small subset of SOG OA neurons to effect appropriate male action patterns. To eliminate Fru^M^ function in OA neurons, we used the *dTdc2-Gal4* line to drive expression of an RNA-mediated interference transgene (*UAS-fru^M^IR*) [29]. This method completely eliminated Fru^M^ expression in the pair of OA ventral paired median neurons (arrow, compare Fig. 2B and C) and in a third ventral unpaired median neuron (compare Fig. 2D and E, *n = 12*). Fru^M^ expression in non-OA/TA neurons was unaffected (data not shown). *dTdc2-Gal4/UAS*-*fru^M^IR* progeny were grown at 29° to ensure elimination of Fru^M^ expression [29] and all subsequent behavioral assays were carried out at 25° with 3–5 day old males. Fru^M^ expression did not return to detectable levels after either the 10-minute courtship choice assay or the 1.5 h aggression assay (data not shown).

#### Courtship choice behavior

As in males with activated OA neurons, the total percentage of time spent performing wing extensions was unchanged between control males and males without Fru^M^/OA neuron function [*UAS*-*fru^M^IR*/+, 17.88±1.53 (±SEM) (n = 12), *dTdc2-Gal4/UAS*-*fru^M^IR*, 17.34±1.9 (n = 17); *P*>0.99, *t* test for independent samples). *dTdc2-Gal4/UAS*-*fru^M^IR* males had a longer latency to initiate courtship toward the female but copulation rates were not significantly different from each other (Table S1). However, in competitive courtship assays, males without Fru^M^ function in OA neurons courted the second male a significantly higher percentage of time than control males. Control males (*UAS*-*fru^M^IR*/+) directed wing extensions/singing to another male 6.6% of the total courtship time, whereas males that lack Fru^M^ function in OA neurons, courted either control males (*UAS*-*fru^M^IR*/+, n = 10) or males of the same genotype (*dTdc2-Gal4*/*UAS*-*fru^M^IR*) 18% and 16.5% of the time respectively (Fig. 2F)

To test whether removing both OA and Fru^M^ function had an additive effect on male behavioral choice defects, we generated males carrying a null mutation in the *Tyramine β-hydroxylase (Tβh^nM18^)* (flies that produce no detectable OA [44]) and that also contain the *dTdc2-Gal4* and *UAS*-*fru^M^IR* transgenes. The body size of these males was smaller than either controls or *dTdc2-Gal4/UAS*-*fru^M^IR* males: therefore we paired *Tβh^nM18^ dTdc2-Gal4/UAS*-*fru^M^IR* males only. Males without OA and Fru^M^ also courted second males significantly more than either transgenic (*UAS*-*fru^M^IR*/+, Fig. 2F, n = 10) or genetic controls (*Tβh^M6^*) [27]). The results however were not additive, indicating that removing OA function or changing the sexual differentiation of the Fru^M^ OA neurons had similar effects on the modulation of male courtship object choice.

Significant differences were observed in the average duration of individual bouts of male-male wing extensions. The M/M wing extension duration was longer in males that lack Fru^M^ function in OA neurons (Fig 2G) whether the wing extension was directed toward a control male (1.63 s±0.18 (±SEM)) or a second experimental male (1.39 s±0.10). In addition, the average M/M wing extension duration was significantly increased in males with OA and Fru^M^ function eliminated, *Tβh^nM18^ dTdc2-Gal4/UAS*-*fru^M^IR*, (1.33 s±0.05, Fig. 2G).

#### Aggression

**Figure 3:**
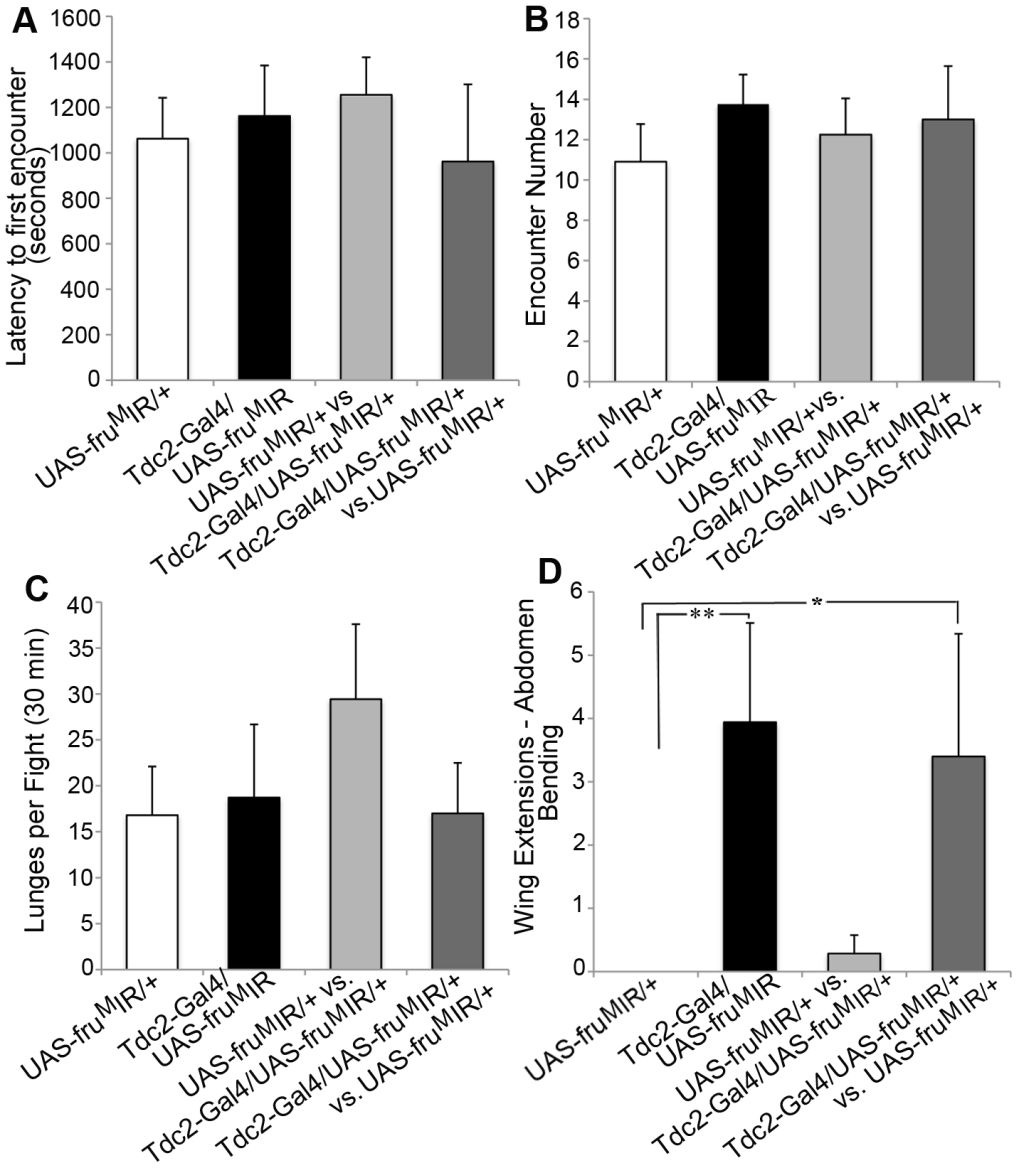
Aggressive behavior is disrupted by courtship patterns in males without Fru^M^ function in OA neurons (A–D). Four separate male pairings in fight chambers were examined. Two control males, *UAS-fru^M^IR/+* (white columns, n = 10), two males without Fru^M^ function in OA neurons, *dTdc2-Gal4/UAS-fru^M^IR* (black columns, n = 17), one control male *UAS-fru^M^IR/+* and one *dTdc2-Gal4/UAS-fru^M^IR* male in which the control male was the dominant male, (light grey column, n = 7), and one control male *UAS-fru^M^IR/+* and one *dTdc2-Gal4/UAS-fru^M^IR* male in which the *dTdc2-Gal4/UAS-fru^M^IR* male was the dominant male, (dark grey column, n = 5). (**A**) Differences in the latency to first encounter between groups were not observed, (*F(3,35) = 0.19*, *P = 0.9*, ANOVA for independent groups). (**B**) Male pairs engaged in similar numbers of encounters in a 30 min fight, (*F(3,35 = 1.59*, *P = 0.2*, ANOVA for independent groups). (**C**) The winning male in each pair performed a similar number of lunges per 30 min fight, (*F(3,35) = 0.4*, *P = 0.75*, ANOVA for independent groups). (**D**) Males that lack Fru^M^ function in OA neurons, *dTdc2-Gal4/UAS-fru^M^IR* males, displayed unilateral wing extensions followed by abdomen bending and attempted copulation whether paired together (black column), or as the dominant or subordinate male when paired with a control male (light and dark grey columns). (A negative binomial model (with log link function) was utilized. A likelihood ratio test of the equality of the group means yielded a highly statistically significant result (P<0.00006). Comparisons of groups yielded statistically significant differences between *dTdc2-Gal4/UAS-fru^M^IR* males male pairings and control *UAS-fru^M^IR/+* males (Bonferroni-adjusted P = 0.0024) and between the dark gray column (one *UAS-fru^M^IR/+*male and one *dTdc2-Gal4/UAS-fru^M^IR* male with the *dTdc2-Gal4/UAS-fru^M^IR* fly establishing dominancy) and control *UAS-fru^M^IR/+*males (white column) (P = 0.0091)).

A previous study reported that OA function is required in a small subset of SOG neurons for *Drosophila* aggression [20]. We generated males carrying the same transgenes described by Zhou et al., 2008 in their studies (*Tdc2-Gal4/UAS-mCD8:GFP*;*Cha-Gal80*) and determined that the OA neurons labeled in these crosses do not express Fru^M^ (Figure S1) and therefore are a separate OA neuronal subset. To determine if the Fru^M^/OA neuronal subset also plays a role in modulating aggression we tested *dTdc2-Gal4/UAS*-*fru^M^IR* males in the fight chamber and found that all components, including latency to fight, encounter number, and numbers of lunges by winners were similar to wildtype (Fig. 3A–C).

The pairing of males in fight chambers without a female target should lead solely to displays of aggressive behavior. Instead, in this context as well, we find males that lack Fru^M^ function also display increased unilateral wing extensions followed by abdomen bending and attempted copulation directed at second males (Fig 3D, Movie S5). *dTdc2-Gal4/UAS*-*fru^M^IR* males perform this behavioral pattern either towards control males (*UAS*-*fru^M^IR*/+) or to other *dTdc2-Gal4/UAS*-*fru^M^IR* males, suggesting again that the selective manipulation of Fru^M^ in SOG OA neurons does not change the perception of external male sensory cues (see also [27]). As in the fights between males with activated OA neurons, both ultimate dominant and subordinate males perform unilateral wing extensions followed by attempted copulation. These results suggest that OA neurons without sex-specific designation through Fru^M^ function also show reduced efficiency in modulating portions of the aggressive vs. reproductive circuitry.

### Detailed anatomy of individual Fru^M^/OA neurons

**Figure 4:**
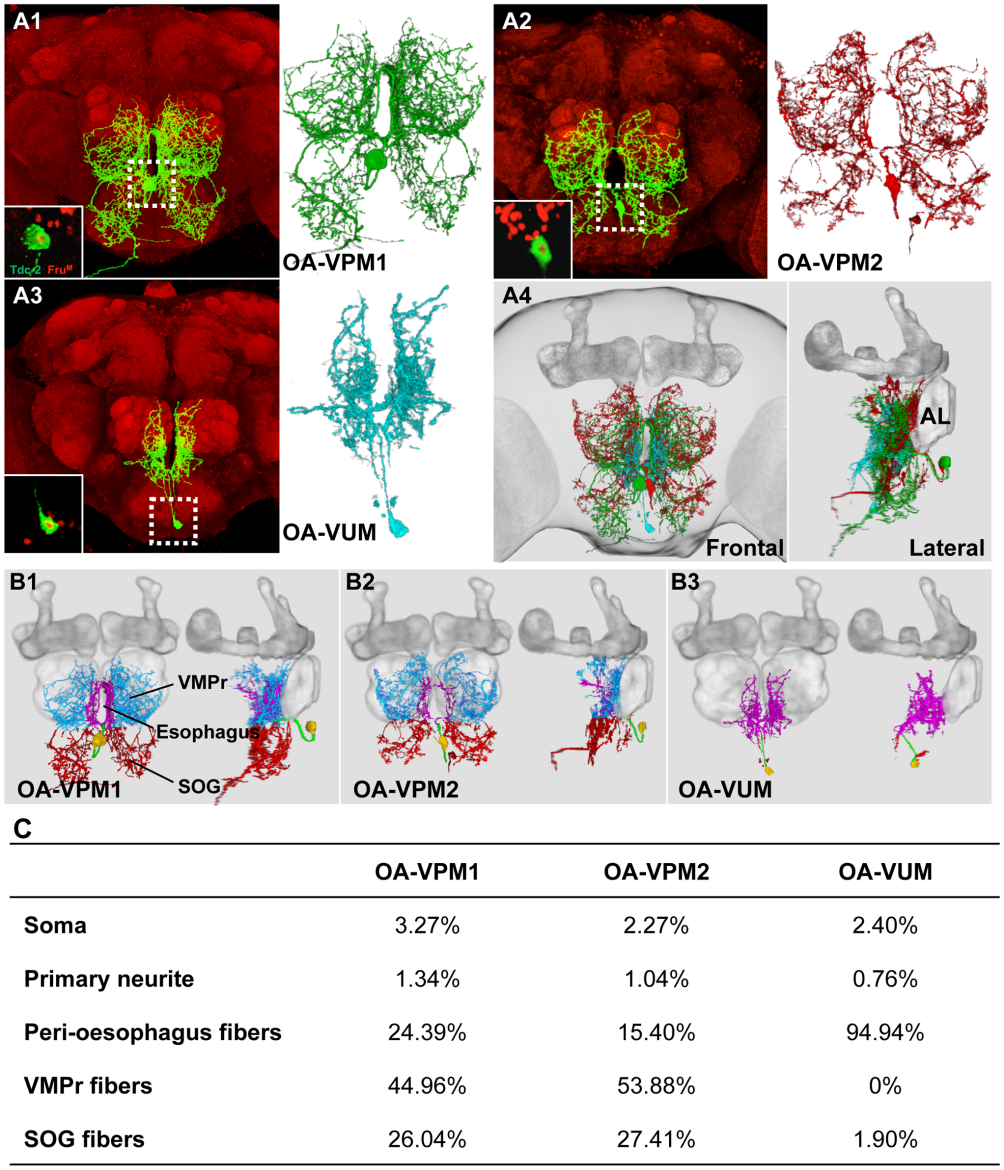
Morphological characterization of Fru^M^/OA neurons. Representative images of Fru^M^/OA neurons in the suboesophageal ganglion. (**A1–A3**) The morphology of three individual Fru^M^/OA neurons, OA-VPM1, OA-VPM12 and OA-VUM. The inset in each picture is an optical slice showing the anti-Fru^M^ positive nucleus within the GFP labeled dTdc2 cell body. (**A4**) Spatial relationships among the three mapped Fru^M^/OA neurons are indicated by simultaneous 3D visualization in the model brain. (**B**) The innervations patterns of the three representative Fru^M^/OA neurons. Fibers from each neuron were segmented based on their innervations of different neuropil regions: soma (orange), primary neurite (green), peri-oesophagus (magenta), VMPr (light blue), and SOG (red). (**C**) Quantitative analysis of the innervation patterns of Fru^M^/OA neurons. The percentage of innervation was determined by counting the numbers of voxels in each neuropil region and dividing that number by the total voxels occupied by the neuron.

Two patterns of behavior typical of male flies, aggression and courtship, are influenced in subtle but definable ways by manipulating the small number of Fru^M^/OA neurons found in the *Drosophila* adult SOG region. In courtship assays, mutant males direct more wing extension/singing behavior towards other males than do control flies. In aggression assays, mutant males insert parts of the courtship ritual (singing and attempted copulation) in an experimental situation in which only aggression should be seen. The small numbers of Fru^M^ OA neurons involved in these actions suggest that this might be an excellent experimental situation in which to examine the neuronal geometry of these functionally relevant neurons. Using standard *Drosophila* genetic tools and recently developed imaging software, we carried out studies examining the morphological profiles of the individual Fru^M^/OA neurons. To visualize the arborizations of these neurons, we used the FLP-out technique [6], [45] and recovered multiple preparations in which single Fru^M^/OA neurons were labeled (Fig. 4A1–A3). These were superimposed on a standard Drosophila brain (Fig. 4A4, B1–3). Two of the Fru^M^/OA neurons are ventral paired median neurons (OA-VPM, nomenclature from [46]), the third is an OA-VUM (OA-ventral unpaired median neuron). As reported in recent studies [39], [41], we also found that each of the Fru^M^/OA neurons project to multiple brain neuropil regions (Fig. 4 and Movie S7).

A detailed volumetric analysis allows quantification of the primary neurite and projection patterns of each of the Fru^M^/OA neurons (Figure 4C). The cell bodies of the paired Fru^M^ OA-VPM neurons are located anteriorly in the SOG and the primary projection patterns from these neurons are mirror-symmetric within the supraesophageal gangion (SPG) and SOG. For the VPMs, the principle area of innervation resides in the ventromedial protocerebrum (vmpr) followed by projections within the SOG and in the group of peri-esophageal fibers (Fig. 4A1,A2,B1,B2,C). For the VUM, the main innervation is in the periesophageal region (Fig. 4C). The ventromedial protocerebrum contains the neuropil structure called the lateral accessory lobe [46] that is involved in polarized light signaling [47]. The lateral accessory lobes are also major targets of central-complex outputs and communicate through ascending and descending neurons with the ventral nerve cord [48]. Neurons of the lateral accessory lobe in the moth *Bombyx mori*, are physically linked and function to collect pheromone-processing information from both sides of the brain [49]. A secondary neurite from the OA-VPM1 neurons can be observed in Figure 4B1 to be descending from the ventrolateral esophagus through the cervical connective (data not shown) and into the thoracic ganglion suggesting a potential mechanism for integrating circuit regulation with motor output.

By far, the greatest density of innervation from the third Fru^M^/OA neuron, OA-VUMd3, is in the area surrounding the esophageal (oes) foramen (Figure 4A3,B3,C). To allow visualization of the arborization patterns of each of the Fru^M^/OA neuron in three-dimensions, we incorporated the data generated from the confocal image stacks of each neuron onto a single standard brain and generated a rotating projection (Figure 4A4, B1–3 and Movie S7). Visualizing the arborizations on a single brain demonstrates that each of the Fru^M^/OA neurons show certain non-overlapping, distinct features. Further explorations of the structure of the Fru^M^/OA neurons focusing on the input and output sources for each of the cells should provide valuable information on how modulatory neurons of this type integrate and disseminate multiple sources of information in biasing patterns of behavioral usage. Of particular interest in future studies will be whether unique morphological features account for the behavioral consequences of changing the sex of these neurons.

## Discussion

Situations requiring rapid decision-making in response to continually changing environmental cues occur repeatedly in nature. In particular, coordinating the expression of disparate behaviors such as predatory vs. defensive behaviors and aggression vs. courtship behaviors can be crucial to the survival and reproductive success of an individual. Extrinsic and intrinsic factors along with experience define the usage of these behaviors by organisms. In *Drosophila*, when a male is placed in an experimental arena with a single female present, robust courtship behavior ensues [50], [51], [52]. If a second male is introduced into the same arena, males usually continue to vigorously court the female and ignore the other male. If, however, a male is placed with a second male in a larger fight chamber containing a resource rich territory (food, potential mates), the same male now engages in vigorous aggressive behavior. Understanding the neural basis of such context-dependent behavioral decision-making remains a critical area of neurobiological investigation.

### Altering the function of OA neurons enhances male-male courtship behavior

In earlier studies, we demonstrated that in environments that favor aggression and/or courtship choice, male flies with reduced levels or with no OA show enhanced levels of male-male courtship [27]. Here we take a different experimental approach by now activating OA neurons through expression of the thermosensitive *Drosophila dTrpA1* ion channel, rather than by eliminating OA function. We find, once again, that courtship behavior directed toward another male fly is significantly increased. How can lowering OA levels via reducing the activity of its biosynthetic enzyme and increasing the availability of OA via increasing the firing of neurons containing the amine, yield the same behavioral phenotype? One possibility for the observed male-male courtship is that either without OA or with activated OA neurons, the dynamic range of OA signaling has been decreased. Specific neural circuits, including the networks that activate courtship behavior, need to function adaptively according to the changing state of the environment or the changing state of the organism. If the courtship circuit cannot be reconfigured or attenuated, the resulting behavioral output may not be reshaped in appropriate ways. The importance of maintaining a dynamic balance between neurotransmitters and neuromodulators in fine tuning neuronal network output and thereby the behaviors that result from their activation, has been studied extensively in numerous systems (reviewed in [53]).

In a crustacean model of aggression, raising or lowering 5HT levels by pharmacological means enhances aggression [54]. Raising or lowering cAMP levels (a usual signaling component of neuromodulation) by genetic means, results in parallel deficits in *Drosophila* olfactory learning and memory [55], [56]. Recent experiments using an intact neuronal circuit in *Drosophila* demonstrate that octopamine and dopamine (DA) bidirectionally modulate intracellular cAMP levels in spatially distinct patterns in adult mushroom body neurons [19]. The balance of neuromodulatory effects of dopamine (DA) and norepinephrine also have been examined in humans and other primates with both amines exhibiting striking, inverted U influences on prefrontal cortex cognitive function (reviewed in [57]). Experiments in vertebrates offer indications of the complexity of neuromodulatory signaling as the beneficial effects of NE and DA receptor stimulation arise from opposing effects on cAMP intracellular signaling pathways; NE α2A receptor binding inhibits [58] while DA-D1 receptor binding activates [59] these pathways. In *Drosophila*, different OA/TA receptors themselves can either stimulate or inhibit the production of cAMP [60]. Results presented here indicate that exploration of the functional roles of *Drosophila* octopaminergic neurons provide a useful system to ask how neuromodulatory inputs at the cellular level alter the properties of a social behavior network. Due to the complexity of second messenger signaling, receptor distribution, and network physiology, understanding the role of individual or small subsets of neuromodulatory neurons will be essential in any system.

### Selective OA neuron function

Although amine neurons are found in relatively small numbers in all species of animals (usually less that 0.5% of the total neuronal population), their fields of innervation are wide reaching essentially all areas of the neuraxis via extensive arbors of processes and broad fields of innervation [61], [62], [63], [64]. The terminal processes of these neurons include traditional appearing synaptic contacts as well as varicosities without underlying synaptic specializations typical of local hormone action [65], [66], [67]. Despite the grouping of amine neuron populations in earlier functional studies, it is becoming increasingly clear that at a small subset or individual neuron level, considerable specificity exists in the fields of innervation of amine neurons and in their function.

Anatomically, evidence for this idea has been presented in detailed morphological studies of *Drosophila* amine neurons [39], [68], [69]. For example, Mao and Davis (2009) report that eight different types of dopaminergic neurons innervate the mushroom bodies of the brain. These authors suggest further that each of the eight might: respond to different input signals; target different areas of the mushroom body; and function to regulate different aspects of behavior. Other recent studies indicate that unique functionality can be assigned to additional small subsets of dopaminergic neurons including: influencing aversive reinforcement and appetitive memory output in *Drosophila* [17], [70], and generating distinct ‘positive’ and ‘negative’ signals in response to reward stimuli in primates [18].

Here we focused on the role of three OA neurons that express the male-specific forms of Fruitless on modulating sex-specific behavioral choice. *fruitless* is a key component of the sex determination pathway encoding sex-specific and non-sex-specific proteins that are members of the BTB-Zn finger transcription factor family [42], [71]. Studies on Fru^M^ function and circuitry have led to the proposal that sensory input, internal states, and individual experience converge onto a sex-specific ‘decision center’ to guide action selection [72]. There is currently little information on how large an influence one might expect then from manipulating the function of single aminergic modulatory neurons in complex behavioral situations. Here, by eliminating Fru^M^ function in a small subset of SOG octopaminergic neurons, we obtained male flies who directed courtship behavior toward other males significantly more than did control males in contexts in which male-female courtship or male-male aggression were anticipated. The normal variety of behavioral patterns concerned with courtship and aggression were retained: only courtship target selection decisions under the specified experimental situations were compromised. By contrast, in chaining assays (groups of males) or single choice assays (male to male) males without Fru^M^ function in OA neurons (Certel, data not shown) or males without OA [20] do not exhibit significant amounts of male-male courtship behavior. The results, therefore, suggest that the function of Fru^M^ in these three particular OA neurons is relevant to male flies in guiding their behavior in specific context-dependent choice situations.

### Detailed anatomy of Fru^M^/OA neurons

The identification of 27 different types of octopaminergic neurons based on morphological features raises the possibility that each neuron or small subsets of neurons regulate unique aspects of OA function [39]. The three-dimensional studies reported here detailing the anatomical structure of the three Fru^M^/OA neurons found in the SOG, also reveal key differences in the shapes and target areas of single neurons within this small subpopulation. The arborization projections of each Fru^M^/OA neuron are extensive and target multiple brain regions providing, as in other insect species, a complex scaffold through which to modulate behavior [73], [74], [75]. Branches of the two Fru^M^-positive OA-VPM1 neurons terminate bilaterally in the ventrolateral protocerebra and the SOG. The SOG receives key contact gustatory pheromone information believed, among other roles, to facilitate sex and species discrimination [76], [77]. Behaviorally, a role in suppressing male-male courtship has been reported for two gustatory receptor genes, Gr32a and Gr33a [78], [79] and axonal projections of Gr32a-expressing neurons terminate both in the SOG and in the ventrolateral protocerebrum [78], [80]. Our anatomical studies demonstrate that the three FruM/OA neurons do not arborize in the major olfactory sensory centers (antennal lobe, mushroom body, and lateral horn). This suggests that olfaction and/or mis-recognition of olfactory cues is not likely to be the cause of the enhanced male-male courtship behavior observed in the behavioral choice assays. Moreover we do not see enhanced male courtship behavior if only males are present in an arena, as in a chaining assay. Such observations suggest that the results obtained here might be best interpreted in a decision-making context.

How might both OA and Fru^M^ function in decision-making during male social interactions? One possibility would be that the Fru^M^/OA neurons regulate sensory inputs to the SOG concerned with sexual recognition. That modulatory neurons can serve such roles is illustrated in a recent study in the medicinal leech demonstrating that the choice between two qualitatively different behaviors is based on a serotonin-mediated presynaptic inhibition of synaptic output from sensory neuron terminals [81]. Another possibility would be that male and female forms of particular neurons (as influenced by Fru^M^ expression) display sex-specific circuitry connections or differ in sex-specific physiological properties in ways that can alter function (e.g., release different amounts of transmitter, express a different co-transmitter, etc.) [72], [82]. Our present morphological and behavioral studies examining this small Fru^M^/OA neuronal population provide necessary refinement steps towards understanding how individual neuromodulatory neurons interact with neuronal circuitry to bias behavioral output.

## Materials and Methods

### Fly stocks

The following strains were used in this study: the *dTdc2-Gal4* line [30], a second remobilized *dTdc2-Gal4#1* line used as a transgenic insertion control [27], the *UAS-dTrpA1* line (II and III chromosomal insertion, [28]), and the *UAS-fru^M^IR/CyO;UAS- fru^M^IR* line [29]. As specified in the text, control males were generated by crossing Canton S females with males from either the *UAS-dTrpA1* or *UAS-fru^M^IR/CyO*;*UAS- fru^M^IR* lines. To generate males that lack OA and Fru^M^ function, the II chromosome *dTdc2-Gal4* line [30], was remobilized onto the X chromosome. The Gal4 driven expression of GFP in OA/TA neurons was verified and then this insertion was recombined onto the *w^−^ Tβh^nM18^* chromosome [44]. Recombinant chromosomes were screened for female sterility (*Tβh^nM18^* mutation) and eye color. The absence of OA production in candidate recombinant lines was verified by HPLC.

### Behavioral Assays

Flies were raised on standard cornmeal medium and kept on a 12-h/12-h day/night cycle in 50% relative humidity. Control and experimental flies carrying the *UAS-dTrpA1* transgene were raised at 19°C until 15 min prior to the beginning of either aggression or courtship assays, and then transferred to 26.5°C at which temperature the assays were performed. In aggression assays, 30 min of recorded fights were scored for aggressive and courtship behavioral patterns. In competitive courtship assays, 10 min following the first wing extension was scored to generate the percentage of time spent performing wing extensions and male-female or male-male courtship. Control and experimental flies carrying the *UAS- fru^M^IR* transgenes were raised at 29°C according to [29] and then shifted to 25°C for either the aggression or competitive courtship assay. Socially naïve adults were collected, painted and aged as previously described [27]. All flies were 3–5 days old at the time of testing, and each pair of flies used in the courtship or aggression assays were the same age and size matched prior to pairing. The aggression and courtship assays were performed as previously described [27], [34].

### Immunohistochemistry

Staged adult male dissected brains were labeled using a previously described protocol [83]. Brains dissected from *hs-FLP*;*dTdc2-Gal4:UAS<CD2*, *y+>CD8-GFP* males for single neuron clonal analysis were labeled with primary antibodies: rat anti-CD8 (1∶300) (Invitrogen, Carlsbad, CA), rabbit anti2-Fru^M^ (1∶3000)[84], and mAb nc82 (1∶100) [85].Secondary antibodies include Alexa Fluor 488-, Alexa Fluor 594- and Alexa Fluor 647-conjugated cross-adsorbed antibodies (Jackson ImmnoResearch Laboratories, West Grove, PA).

### Anatomy and 3D Image Reconstruction

Brain samples were cleared in *FocusClear™* (CelExplorer, Taiwan) for 5 min, mounted in a drop of *MountClear™* (CelExplorer, Taiwan) and then imaged under a Zeiss LSM 510 confocal microscope [86]. Single neurons labeled by FLP-out GFP expression were digitally segmented and compiled onto the established standard mushroom body (MB) as a common 3D framework using the nc82 immunolabeled MB in the sample as a reference [87]. Spatial distribution of each individual neuron in relation to different brain regions was first visually inspected and then quantified with *Amira* (Visage Software, San Diego, CA).

### Statistical Analyses

Normally distributed data were analyzed by using Student's *t* test. For data sets that were not normally distributed, means were statistically compared using the Wilcoxon rank sum test for nonparametric data. For the data sets in Figures 1 and 3 that measured wing extensions, Poisson regression and related models were considered. The group variances were generally much larger than the corresponding means and the relationship was not linear, suggesting Poisson and quasi-Poisson models were not appropriate. A negative binomial model (with log link function) provided a very good fit by multiple measures and was utilized. Statistical analyses were performed with JMP IN software, Version 8 (SAS Institute, Cary, NC), VasserStat (http://faculty.vassar.edu/lowry/VassarStats.html) or the R project software (http://www.r-project.org/).

## Supporting Information

Table S1 (0.13 MB JPG)

Figure S1 A subset of OA neurons implicated in controlling aggression does not express FruM. Confocal sections of a transgenic dTdc2-Gal4/UAS-mCD8:GFP;Cha-Gal80 adult male brain labeled with anti-GFP (green) and anti-FruM antiserum (red). The SOG OA neurons expressing GFP (arrow) do not express FruM.
(0.68 MB TIF)

Movie S1 UAS-dTrpA1/+ Courtship Two UAS-dTrpA1/+ males with one Canton S female in the competitive courtship choice assay. The movie depicts the male-female courtship behavior that is performed with the control +/UAS-dTrpA1 males. All courtship behavior is directed toward the female.
(1.87 MB MP4)

Movie S2 Tdc2-dTrpA1 Courtship Example 1 Two Tdc2-Gal4/UAS-dTrpA1 males with one Canton S female in the competitive courtship choice assay. Male-male courtship is observed in this clip. The male-male courtship continues even in close proximity to the female and with male rejection behavior.
(1.42 MB MP4)

Movie S3 Tdc2-dTrpA1 Courtship Example 2 Two Tdc2-Gal4/UAS-dTrpA1 males with one Canton S female in the competitive courtship choice assay. Male-male courtship with tracking, orienting and abdomen bending is observed in this clip.
(2.79 MB MP4)

Movie S4 Tdc2-dTrpA1 Aggression Two Tdc2-Gal4/UAS-dTrpA1 males in the fight chamber. The male painted white extends a unilateral wing throughout the clip. The wing extensions that are scored include vibration followed by abdomen bending. There are three examples of vibrating unilateral wing extension with abdomen bending in this clip.
(4.90 MB MP4)

Movie S5 Tdc2-FruIR Aggression Two dTdc2-Gal4/UAS-fruMIR males in the fight chamber. The male painted white first lunges at the second male then performs a unilateral wing extension with vibration followed by abdomen bending. Six lunges by the white-painted male follow the wing extension-abdomen bending pattern.
(0.75 MB MP4)

Movie S6 UAS-dTrpA1/+ vs. Tdc2-dTrpA1 Clip 13 Aggression One UAS-dTrpA1/+ male (white paint) lands on the food territory in the fight chamber and performs seven total lunges and wing flicks toward the second dTdc2-Gal4/UAS-dTrpA1 (black paint) male ultimately leading to the retreat of the dTdc2-Gal4/UAS-dTrpA1 male.
(2.08 MB MP4)

Movie S7 Rotating projection of the individual FruM/OA neurons generating from confocal image data of each neuron onto a single standard brain.
(14.87 MB MPG)
