# Supplementary figures and images for: Octopamine Neuromodulatory Effects on a Social Behavior Decision-Making Network in Drosophila Males
Source: PLoS One. 2010 Oct 12;5(10):e13248. doi: 10.1371/journal.pone.0013248 (PMC2953509; doi:10.1371/journal.pone.0013248)

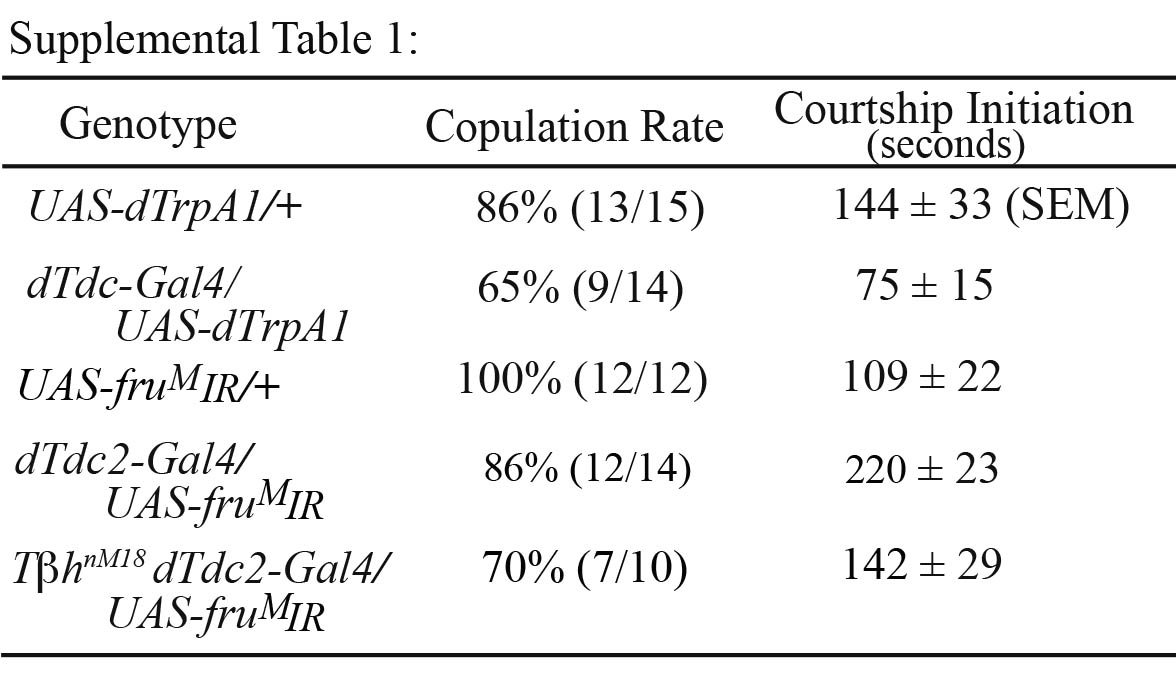

Supplement: Table S1 — (0.13 MB JPG) [file pone.0013248.s001.jpg]

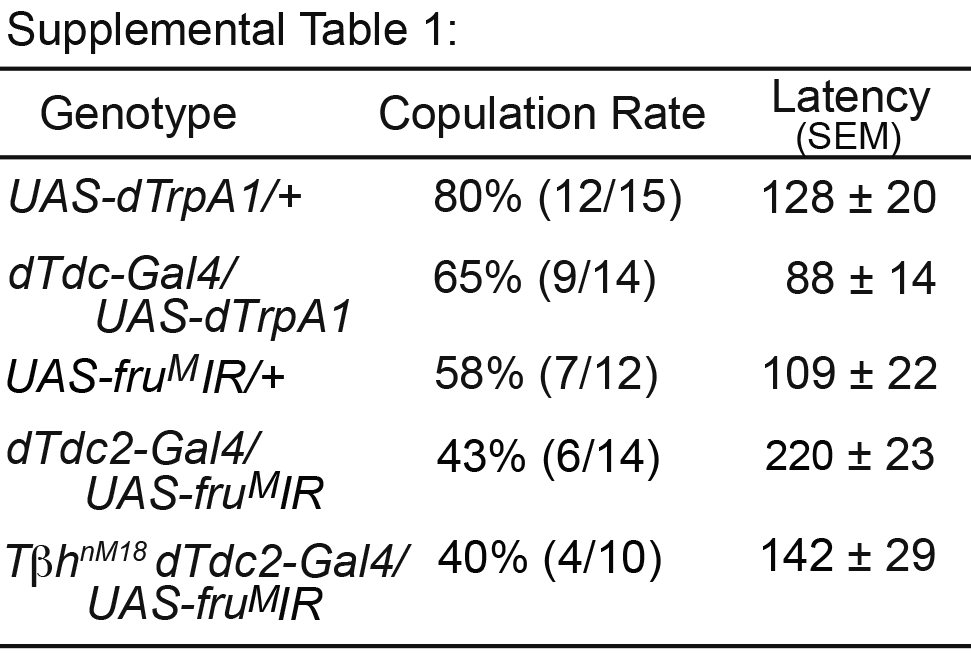

Supplement: Figure S1 — A subset of OA neurons implicated in controlling aggression does not express FruM. Confocal sections of a transgenic dTdc2-Gal4/UAS-mCD8:GFP;Cha-Gal80 adult male brain labeled with anti-GFP (green) and anti-FruM antiserum (red). The SOG OA neurons expressing GFP (arrow) do not express FruM. (0.68 MB TIF) [file pone.0013248.s002.tif]
